# CO-FLOW: COvid-19 Follow-up care paths and Long-term Outcomes Within the Dutch health care system: study protocol of a multicenter prospective cohort study following patients 2 years after hospital discharge

**DOI:** 10.1186/s12913-021-06813-6

**Published:** 2021-08-21

**Authors:** L. Martine Bek, Julia C. Berentschot, Merel E. Hellemons, Susanne M. Huijts, Joachim G. J. V. Aerts, Jasper van Bommel, Michel E. van Genderen, Diederik A. M. P. J. Gommers, Gerard M. Ribbers, Majanka H. Heijenbrok-Kal, Rita J. G. van den Berg-Emons, the CO-FLOW Collaboration Group, L. Martine Bek, Julia C. Berentschot, Merel E. Hellemons, Susanne M. Huijts, Joachim G. J. V. Aerts, Majanka H. Heijenbrok-Kal, Rita J. G. van den Berg-Emons, Gerard M. Ribbers, Jasper van Bommel, Michel E. van Genderen, Diederik A. M. P. J. Gommers, Erwin Ista, Robert van der Stoep, Rutger Osterthun, Markus P. J. M. Wijffels, Jorrit Slaman, Marieke M. Visser, Janette J. Tazmi-Staal, Eva G. Willems, Roxane Heller, Shai A. Gajadin, Wouter J. B. Blox, Laurien Oswald, Sieshem Bindraban, Rob Slingerland, Herbert J. van de Sande, Hawre Kadir, Marc van Lanen, Ronald N. van Rossem, Stephanie van Loon-Kooij

**Affiliations:** 1 Department of Rehabilitation Medicine, Erasmus MC, University Medical Center Rotterdam, Rotterdam, The Netherlands; 2 Department of Respiratory Medicine, Erasmus MC, University Medical Center Rotterdam, Rotterdam, The Netherlands; 3 Department of Adult Intensive Care Medicine, Erasmus MC, University Medical Center Rotterdam, Rotterdam, The Netherlands; 4 Rijndam Rehabilitation, Rotterdam, The Netherlands

**Keywords:** COVID-19, Aftercare paths, Physical recovery, Cognitive recovery, Psychosocial recovery, Participation, Rehabilitation, Satisfaction, Barriers, Facilitators

## Abstract

**Background:**

First studies indicate that up to 6 months after hospital discharge, coronavirus disease 2019 (COVID-19) causes severe physical, cognitive, and psychological impairments, which may affect participation and health-related quality of life (HRQoL). After hospitalization for COVID-19, a number of patients are referred to medical rehabilitation centers or skilled nursing facilities for further treatment, while others go home with or without aftercare. The aftercare paths include 1] community-based rehabilitation; 2] in- and outpatient medical rehabilitation; 3] inpatient rehabilitation in skilled nursing facilities; and 4] sheltered care (inpatient). These aftercare paths and the trajectories of recovery after COVID-19 urgently need long-term in-depth evaluation to optimize and personalize treatment. CO-FLOW aims, by following the outcomes and aftercare paths of all COVID-19 patients after hospital discharge, to systematically study over a 2-year period: 1] trajectories of physical, cognitive, and psychological recovery; 2] patient flows, healthcare utilization, patient satisfaction with aftercare, and barriers/facilitators regarding aftercare as experienced by healthcare professionals; 3] effects of physical, cognitive, and psychological outcomes on participation and HRQoL; and 4] predictors for long-term recovery, health care utilization, and patient satisfaction with aftercare.

**Methods:**

CO-FLOW is a multicenter prospective cohort study in the mid-west of the Netherlands with a 2-year follow-up period. Measurements comprise non-invasive clinical tests and patient reported outcome measures from a combined rehabilitation, pulmonary, and intensive care perspective. Measurements are performed at 3, 6, 12, and 24 months after hospital discharge and, if applicable, at rehabilitation discharge. CO-FLOW aims to include at least 500 patients who survived hospitalization for COVID-19, aged ≥18 years.

**Discussion:**

CO-FLOW will provide in-depth knowledge on the long-term sequelae of COVID-19 and the quality of current aftercare paths for patients who survived hospitalization. This knowledge is a prerequisite to facilitate the right care in the right place for COVID-19 and comparable future infectious diseases.

**Trial registration:**

The Netherlands Trial Register (NTR), https://www.trialregister.nl. Registered: 12-06-2020, CO-FLOW trialregister no. NL8710.

## Background

From the beginning of 2020 the world has been overwhelmed by coronavirus disease 2019 (COVID-19), a new respiratory infectious disease that was first discovered in China at the end of 2019. Hospitalization, including Intensive Care Unit (ICU) treatment, is frequently needed. Hospitalized patients have to deal with mild to severe illness, often without the support of family and loved ones. This new disease and surreal situation are expected to cause severe and long-lasting physical, cognitive, and psychological sequelae, affecting participation and health-related quality of life (HRQoL). Little is known yet about the long-term sequelae of COVID-19, and therefore, the European Academy of Rehabilitation Medicine warned for the unknown aftershocks of the pandemic and called for action [1].

Many factors will play a role in the potentially severe long-term sequelae after hospital discharge. Since recent years it has come to the attention that ICU treated patients can experience a combination of long-term physical, cognitive, and psychological sequelae, known as the Post-Intensive Care Syndrome [2, 3]. COVID-19 has unique features in that ICU length of stay is relatively long and that patients are frequently immobilized in prone position with (high pressure) mechanical ventilation [4]. After ICU, patients with COVID-19 can experience musculoskeletal, neurological, and psychological impairments [5–7]. Furthermore, the viral pneumonia may cause permanent lung injury. Secondarily, ventilator induced damage will occur in a large proportion of patients, leading to permanent pulmonary function decline, necessitating respiratory rehabilitation or even long-term oxygen support [8]. A large proportion of the severely ill patients experiences thrombotic complications such as pulmonary embolism or ischemic brain infarction, which can result in long-term morbidity (e.g. chronic thrombo-embolic pulmonary hypertension, and cognitive and motor impairments) [9].

Although long-term sequelae of COVID-19 are expected to be most prominent in ICU treated patients, also hospitalized patients without ICU treatment may experience long-term impairments in several areas [10–12]. Likewise, many of these patients are severely ill, are immobilized for a relatively long period, may experience complications, are often restricted in having visitors, are confronted by isolation measures, and may develop permanent pulmonary damage. Such a unique situation could, besides affecting physical function [13], potentially result in cognitive and psychological impairments such as anxiety, concentration problems, and post-traumatic stress [14].

Rehabilitation, with its multidisciplinary approach (rehabilitation medicine, physical/sports therapy, occupational therapy, psychology, social work, dietetics, speech/language therapy), is the cornerstone of management of the consequences of COVID-19. Minimizing the effects of potential long-term impairments on participation (including return to work, leisure activities, and social relationships) and HRQoL are essential rehabilitation goals.

The sudden COVID-19 pandemic forced quick development of aftercare paths for this new patient group. These aftercare paths comprise 1] community-based rehabilitation; 2] in- and outpatient medical rehabilitation; 3] inpatient rehabilitation in skilled nursing facilities; and 4] sheltered care (inpatient). However, whether these newly developed aftercare paths provide patients with the right care in the right place is unknown yet. For instance, the trajectory of recovery after hospitalization for COVID-19 and its predictors remain unknown to date, and outcomes beyond 6 months are still scarce [11, 15–18]. Studies in survivors from other coronavirus pneumoniae (severe acute respiratory syndrome [SARS] and Middle East respiratory syndrome [MERS]) suggest long-term sequelae lasting for months or even years [19–22]. Early studies in COVID-19, mostly with a cross-sectional design, indicate that a broad range of sequelae may occur up to 6 months after hospitalization, varying from pulmonary impairments and residual radiological abnormalities to impairments in cardiorespiratory and neuromuscular fitness, and symptoms such as dyspnea, fatigue, anxiety, depression, and sleep disturbances [11, 15, 23, 24]. However, how long these symptoms will last and to what extent recovery will occur is still unclear [25].

Besides in-depth knowledge on the trajectories of physical, cognitive, and psychological recovery, insight in the aftercare paths is needed, such as patient flows across the different paths, healthcare utilization, patient satisfaction with aftercare, and barriers/facilitators regarding aftercare as experienced by health care professionals. This knowledge will further facilitate optimization of the aftercare paths for COVID-19 and comparable future infectious diseases, and will build on the relatively underexamined post-ICU recovery and effectiveness of rehabilitation [26].

Given the above, the newly developed aftercare paths for COVID-19 urgently need to be evaluated [27]. Evaluation should be performed prospectively in the short, medium, and long term, on a broad range of sequelae and predictors, participation, HRQoL, and aftercare paths. CO-FLOW, with its combined rehabilitation, pulmonary, and ICU perspective, offers this holistic and long-term (2 years) follow-up approach.

### Aims

The aim of the CO-FLOW study is to gain in-depth knowledge on the long-term sequelae in patients who survived hospitalization for COVID-19 and to further develop the aftercare paths for COVID-19 and other comparable future infectious diseases. More specifically, CO-FLOW aims, by following the outcomes and aftercare paths of all COVID-19 patients after hospital discharge, to systematically study over a 2-year period: 1] trajectories of physical, cognitive, and psychological recovery; 2] patient flows, healthcare utilization, patient satisfaction with aftercare, and barriers/facilitators regarding aftercare as experienced by healthcare professionals; 3] effects of physical, cognitive, and psychological outcomes on participation and HRQoL; and 4] predictors for long-term recovery, health care utilization, and patient satisfaction with aftercare.

## Methods

### Eligibility criteria

All COVID-19 patients who survived hospitalization in one of the hospitals in the Rotterdam-Rijnmond-Delft region, which is in the mid-west of the Netherlands, are eligible if they fulfill the following in- and exclusion criteria. Inclusion criteria: 1] COVID-19 diagnosis (based on positive polymerase chain reaction or multidisciplinary team decision based on symptoms and computed tomography (CT) or positive serology); 2] requiring and surviving hospitalization; 3] within 6 months (but preferably within 3 months) after hospital discharge; 4] patient or relative has sufficient knowledge of Dutch or English language. Exclusion criteria: 1] age < 18 years; 2] incapacitated subjects.

### Sample size

A formal sample size calculation was not performed, because we did not focus on a single outcome to base our power calculation on in this multiple outcome prospective cohort study. The original sample size estimation was ≥335 patients, estimated on the number of patients hospitalized in the first wave of the COVID-19 pandemic in the Rotterdam-Rijnmond region. Due to the ongoing COVID-19 pandemic this was later extended to ≥500 patients, including patients from the second and third wave.

### Study design

The CO-FLOW study has a prospective multicenter cohort design, in which outcomes are studied from a combined rehabilitation, pulmonary, and ICU perspective. Participating institutions are hospitals (*n* = 7, among which an academic hospital), a rehabilitation center, a skilled nursing facility, and a sheltered care facility, all in the region Rotterdam-Rijnmond-Delft. Since July 1st 2020, patients are included after hospital discharge and followed until 2 years thereafter throughout the continuous health care chain.

Measurements are performed at 3, 6, 12, and 24 months after hospital discharge. Patients admitted to inpatient medical rehabilitation or to a skilled nursing facility after hospital discharge undergo an additional measurement at discharge. Measurements comprise (non-invasive) clinical tests and patient reported outcome measures (PROMs), and are predominantly part of regular care; Table 1. During study visits, additional non-invasive measurements are performed by a trained researcher or research assistant. These measurements are performed when patients visit their own hospital for their regular follow-up after hospital discharge. The length of this regular follow-up period depends on the severity of disease and is based on the decision of the treating physician. After patients are discharged from regular care, they are invited to visit Erasmus MC, University Medical Center Rotterdam, for the remaining study visits. In case patients are not willing or able to come to Erasmus MC, a research assistant visits them at home to perform the study measurements.

### Outcome measures

Outcome measures concern A] trajectories of recovery; B] predictors from a rehabilitation, pulmonary, and ICU perspective; and C] aftercare paths.

#### A] Trajectories of recovery

##### Physical function

Pulmonary function: spirometry measuring forced vital capacity (FVC), forced expiratory capacity at the first second of exhalation (FEV1), and diffusing capacity of the lung for carbon monoxide adjusted for hemoglobin (DLCOc) results are collected, if tests are performed during aftercare. The Global Lung Function Initiative Network (GLI) reference values were used to express percentages of predicted values, the z-scores and the lower limit of normal (LLN) [28, 29]; Radiographic abnormality: chest X-ray and CT-scan results are collected, if performed during aftercare; Neuromuscular fitness: hand-held dynamometry with Jamar hydraulic dynamometer (Lafayette Instrument Company, USA) to assess maximum grip strength (patient squeezes the dynamometer three times with each hand) [30, 31]; Medical Research Council (MRC) to manually assess muscle strength in the upper and lower limb [32]; Cardiorespiratory fitness: 6-min walk test (6MWT), a submaximal exercise test measuring the distance walked in 6 min over a 30 m (mainly) or 20 m walkway, depending on test location. Secondary outcomes include exercise-induced changes in saturation, heart rate, and perceived fatigue and dyspnea (Borg scale) [33]; 1-min sit-to-stand test (1MSTS) in which the number of successfully standing up from a chair without using hands for support is counted during 1 min [34]; Body mass index (BMI): height and weight measurement; Fat-free mass: arm circumference [35, 36].

Mobility: De Morton Mobility Index (DEMMI), an observation test to assess problems with mobility, balance, movement, and daily activities in the elderly [37].

Physical activity, sedentary behavior, and sleep: GENEActiv wrist watch (Activinsights, Kimbolton, UK), a small and light-weight tri-axial accelerometer. This watch is worn on the dominant wrist for 7 consecutive days to objectively assess physical (in) activity and sleep behavior in daily life.

##### Cognitive function

Subjective: Cognitive Failure Questionnaire (CFQ) to assess the frequency of experienced cognitive failures in everyday life, such as absent-mindedness, slips and errors of perception, memory, and motor functioning [38, 39].

Objective: Montreal Cognitive Assessment (MoCA), a rapid screening instrument for cognitive dysfunction, assessing different domains: visuospatial/executive function, attention, concentration, working memory, language, short-term memory, and orientation [40]. If indicated, patients receive additional cognitive tests as part of regular care only: Location Learning Test (LLT) [41], Trail Making Test (TMT) [42], Stroop Test [43, 44], Letter-Digit Substitution Test (LDST) [45], Digit Span [46], and Letter and Category Fluency [47, 48].

##### Psychological function

Mood: Hospital Anxiety and Depression Scale (HADS), a general measure of emotional distress containing two subscales: anxiety and depression [49].

Post-Traumatic Stress Syndrome: Impact of Event Scale-Revised (IES-R), assessing traumatic consequences that (senior) patients may experience after treatment in the hospital or ICU, comprising the domains of intrusion, avoidance, hyperarousal, and a total subjective stress IES-R score [50].

##### Secondary measures

Independency in activities of daily life is measured with the Barthel Index (BI) [51]; Self-reported physical activity with the International Physical Activity Questionnaire (IPAQ-short form) targeting vigorous, moderate, and light activities [52]; Self-reported physical fitness with the International Fitness Scale (IFIS), a questionnaire assessing general physical fitness, cardiorespiratory fitness, muscular strength, speed-agility, and flexibility [53]; Fatigue with the Fatigue Assessment Scale (FAS), a simple and short self-administered questionnaire to indicate chronic fatigue [54]; Health-related quality of life with the SF-36 and the 5-level EuroQoL-5D (EQ-5D-5L) questionnaires. The SF-36 questionnaire is a multidimensional instrument for measuring general health condition, referring to limitations in functioning due to physical and/or emotional limitations [55]. The EQ-5D-5L consists of the 5-level EQ-5D index (mobility, self-care, usual activities, pain/discomfort, anxiety/depression) and a visual analogue scale (EQ-VAS) [56]; Burden of disease with the Assessment of Burden of Corona (ABC) tool, an innovative tool that measures and visualizes integrated health status. An important part of the tool is the ABC scale, which is largely based on the Clinical COPD Questionnaire, and consists of five domains (symptoms, functional state, mental state, emotions, and fatigue) [57]; Self-reported COVID-19 symptoms with the COVID-19 Symptom Checklist, developed for the purpose of the study, that screens novel symptoms since the onset of COVID-19; Sleep quality with the Pittsburgh Sleep Quality Questionnaire (PSQI) to assess subjective sleep quality, sleep latency, sleep duration, habitual sleep efficiency, sleep disturbances, use of sleeping medication, and daytime dysfunction [58]; Participation with the Utrecht Scale for Evaluation of Rehabilitation-Participation (USER-P), assessing participation in daily life activities in three scales: frequency, restrictions, and satisfaction [59]; and Recovery with the novel Core Outcome Measure for Recovery, assessing the absence of symptoms related to the illness, the ability to do usual daily activities, and the return to state of health as prior to COVID-19 [60].

#### B] Predictors for long-term recovery

Patient characteristics: age, sex, education, marital status, socio-economic status, cultural background, pre-injury employment and living situation, comorbidity, smoking, alcohol use (all questionnaire developed for the purpose of the study), pre-morbid physical activity class (Saltin-Grimby Physical Activity Scale, SGPALS) [61], and coping (Coping Inventory for Stressful Situations, CISS) [62, 63].

Clinical characteristics (electronic patient records): laboratory biomarkers and hyperinflammation (serum creatinine, estimated glomerular filtration rate using the Chronic Kidney Disease Epidemiology Collaboration formula, C-reactive protein, ferritin, alanine aminotransferase, hemoglobin, mean corpuscular volume, thrombocytes, lymphocyte count, D-dimer, NT-proBNP, IL-6, presence of IgG and IgM antibodies for COVID-19), duration of hospital and ICU stay, quantity and type of oxygen supplementation, duration of mechanical ventilation, acute physiology and chronic health (APACHE sII score), severity of pulmonary disease (CO-RADS score CT-scan), thrombosis, tracheostomy, delirium, pulmonary embolism, other complications during hospital stay, readmission and vaccination status, and nutritional status with the Short Nutritional Assessment Questionnaire (SNAQ) [64, 65].

#### C] Aftercare paths

Patient flows: questionnaire developed for purpose of the study and electronic patient records, to investigate patient flows between 1] community-based rehabilitation; 2] in- and outpatient medical rehabilitation; 3] inpatient skilled nursing facility; and 4] sheltered care (inpatient).

Health care utilization: iMTA Medical Consumption Questionnaire (iMCQ) assessing healthcare use [66].

Productivity losses: iMTA Productivity Costs Questionniare (iPCQ) assessing productivity losses [67].

Patient-reported experience measure: Satisfaction with COVID-19 Aftercare Questionnaire (SCAQ), a post-hospitalization questionnaire developed for the purpose of this study in co-creation with ex-COVID-19 patients and their relatives; available upon request.

Organization of COVID-19 aftercare: questionnaire to assess the level of organization of COVID-19 aftercare as perceived by healthcare professionals and to explore barriers and facilitators for aftercare experienced by the professionals. This questionnaire was developed for the purpose of this study and available upon request, based on the Care Process Self-Evaluation Tool [68].

**Table 1:** Overview of study measurements

	Discharge hospital	Discharge inpatient rehabilitation	3 months after discharge hospital	6 months after discharge hospital	12 months after discharge hospital	24 months after discharge hospital
Patient information/informed consent	x	x	*			
Patient characteristics		x	*			
Clinical characteristics		EPR^$^	*			
General questions, health care path, recovery		x	x	x	x	x
Pre-morbid physical activity class (SGPALS)			x			
COVID-19 Symptom Checklist		x	x	x	x	x
**Questionnaires: online or postal mail**						
Independency general daily life activities: BI		□	x	x	x	x
Physical activity: IPAQ-short form			x	x	x	x
Physical fitness: IFIS		x	x	x	x	x
Fatigue: FAS		x	□	x	x	x
Mood: HADS		□	□	x	x	x
Post-Traumatic Stress Syndrome: IES-R		□	□	x	x	x
Cognition: CFQ		x	x	x	x	x
Coping: CISS					x	
Health-related quality of life: SF-36, EQ-5D-5L		□	□/x	x	x	x
Burden of disease: ABC-tool			□	x	x	x
Sleep quality: PSQI		x	x	x	x	x
Participation: USER-P			x	x	x	x
Health Care utilization: iMCQ, iPCQ			x	x	x	x
Patient satisfaction with COVID-19 aftercare: SCAQ					x	
**Clinical tests at hospital, inpatient rehabilitation, or at home**						
Mobility: DEMMI		□	x	x	x	x
Antropometry: body mass index, arm circumference		□	x	x	x	x
Nutritional status: SNAQ		□	x	x	x	x
Cognition: MoCA		□	x	x*	x*	x*
Cognition: TMT, LLT a.o.^&^		□	□		□	
Pulmonary function: spirometry^&^		□	□	□	□	□
Radiographic abnormality: CT-scan^&^, X-ray^&^			□	□	□	□
Neuromuscular fitness: handgrip strength, MRC*		□	x	x*	x*	x*
Cardiorespiratory fitness: 6MWT, 1MSTS		□	x	x	x	x
Physical activity and sleep: accelerometry			x	*	x	x

□ = standard care in rehabilitation center or skilled nursing facility or standard care at regular follow-up in hospital; x = study measurement; ^$^ EPR = Electronic patient record; * if missed or submaximal score at previous assessment; ^&^ If indicated; when patients are discharged from regular follow-up these measurements are not performed

### Data management

Measurements are performed by trained research assistants in the participating centers under supervision of qualified researchers. Data is collected in Castor Electronic Data Capture system (Castor EDC), a cloud based clinical data management platform, and the platform ‘Gezondheidsmeter.nl’. Surveys including all PROMs are sent to patients through Castor EDC via email, facilitating automatic data storage in the database. Clinical data are collected from electronic patient records by research assistants. Also, we use the Erasmus MC COVID Research (EraCoRe) database for collection of outcomes collected during regular care.

### Statistical analyses

#### A] Trajectories of recovery

The course of physical, cognitive, and psychological recovery over time will be analyzed using linear mixed models (LMM) and/or generalized estimating equations (GEE) analysis for outcomes on an interval scale (linear) and for binary outcomes (logistic), respectively, on an intention-to-treat basis. As part of each model, a covariance matrix is estimated that represents the within-subject dependencies in repeated measurements. Level of recovery at each time point will be included as the dependent variable in the LMM and GEE models. Measurement time (3, 6, 12, and 24 months) is entered as independent variable. In post-hoc analyses, significant recovery over total follow-up and from time point to time point (3, 6, 12 and 24 months) will be identified for each outcome. These analyses will reveal whether overall or partial recovery is reached and which disabilities will persist in the long term. Likewise, trajectories of participation and HRQoL will be studied, including the impact of potential physical, cognitive, and/or psychological disabilities on these outcomes.

#### B] Predictors for long-term recovery

For this analysis we will develop multivariable prediction models. LMM/GEE models including repeated measurements will be constructed to identify predictors for long-term recovery. Patient characteristics (e.g. age, sex, cultural background) and clinical characteristics (e.g. length of ICU stay, APACHE II score, duration of mechanical ventilation) will be entered as independent variables to the models, with recovery as dependent variables. Significant predictors (*p* < 0.05) will be included in multivariable models using a Bonferroni correction, dividing the significance level (alpha< 0.05) by the number of predictors included in the model. These models will identify independent predictors for recovery, which may facilitate in preventing or treating unfavorable health outcomes in future patients.

#### C] Aftercare paths

The proportions of patients flows through the different aftercare paths will be calculated. Recovery within and between the different patient flows over time will be analyzed by entering the type of aftercare path as independent variable to the LMM/GEE models. Speed of recovery and (time of) maximum recovery reached will be compared between aftercare paths by studying interactions between paths and time. Comparing trajectories of different aftercare paths and for multiple outcomes may show in which time frames recovery increases most and when it levels off. Models will be adjusted for case mix.

Health care utilization (number of consultations with general practitioner, physiotherapist, occupational therapist, speech and language therapist, psychologist, dietician, pulmonologist, hospitalizations, etc.) will be calculated for each aftercare path. The effect of cumulative treatment time on recovery will be analyzed at each measurement time using the LMM/GEE models. Likewise, effects of diversity on recovery will be studied by adding variables such as age, sex, and cultural background to the LMM/GEE models. These analyses will inform treatment decisions regarding timing, type, and volume of rehabilitation required for optimal outcomes for specific groups of patients. Patient satisfaction with treatments received in each aftercare path and barriers/facilitators experienced by professionals will be studied using descriptive statistics.

## Discussion

### Relevance

The CO-FLOW study will provide in-depth knowledge on the unknown long-term aftershocks of the COVID-19 pandemic and the quality of current aftercare paths for patients who survived hospitalization. This knowledge is indispensable to facilitate optimization and personalization of aftercare for the current pandemic, ensuring the right care in the right place. Furthermore, the CO-FLOW study will deliver an experience- and evidence-based aftercare path for a potential future COVID-19 outbreak and comparable infectious diseases. In addition, this will expand our knowledge on the relatively underexamined post-ICU recovery and effectiveness of rehabilitation.

### Strengths

Strengths of our study are that 1] CO-FLOW comprises long-term (2 year) monitoring after hospital discharge, with a strong focus on clinical tests; 2] CO-FLOW recruits all COVID-19 hospital survivors, both with and without ICU treatment, which is indispensable because long-term impairments are also expected in patients without ICU treatment; 3] patients are followed over all aftercare paths. This also concerns patients who have been discharged from regular care; 4] CO-FLOW applies a holistic approach focusing on physical, cognitive, and psychological outcomes, participation (including return to work, which is an important outcome in the younger patients), HRQoL, and a wide range of predictors of long-term recovery, among which diversity.

### Limitations

Some limitations have to be mentioned: 1] The CO-FLOW study only focusses on a part of the Netherlands, the Rotterdam-Rijnmond-Delft region. This one-region approach facilitates fast implementation of measurement infrastructure and long-term integral monitoring of all patients who survived hospitalization, including patients who have been discharged from regular care. Knowledge, expertise, and infrastructure of the CO-FLOW study is transferable to other regions and countries to facilitate organization and optimization of aftercare; 2] A control group of patients without COVID-19 is not included in CO-FLOW which hampers interpretation of outcomes; 3] For most of our outcome parameters, no pre-morbid information is available. This hampers interpretation of recovery, particularly in patients with co-morbidity.

## Data Availability

Data sharing is not applicable to this article. The datasets and/or metadata that will be generated and/or analyzed during the current study will be made available by using a digital object identifier (DOI) through Data Archiving and Network Services (DANS) https://dans.knaw.nl/en.

## Abbreviations

CO-FLOW: COvid-19 Follow-up care paths and Long-term Outcomes Within the Dutch health care system
COVID-19: Coronavirus disease
NTR: the Netherlands Trial Register
ICU: Intensive care unit
SARS: Severe Acute Respiratory Syndrome
MERS: Middle East Respiratory Syndrome
Erasmus MC: Erasmus University Medical Center
